# Determining the Orientation and Localization of Membrane-Bound Peptides

**DOI:** 10.2174/138920312800785049

**Published:** 2012-05

**Authors:** Walter Hohlweg, Simone Kosol, Klaus Zangger

**Affiliations:** Institute of Chemistry / Organic and Bioorganic Chemistry, University of Graz, Heinrichstrasse 28, A-8010 Graz, Austria

**Keywords:** Antimicrobial peptides, immersion depth, membrane-bound peptides, NMR spectroscopy, orientation, paramagnetic relaxation enhancement, peptide hormones, toxins.

## Abstract

Many naturally occurring bioactive peptides bind to biological membranes. Studying and elucidating the mode of interaction is often an essential step to understand their molecular and biological functions. To obtain the complete orientation and immersion depth of such compounds in the membrane or a membrane-mimetic system, a number of methods are available, which are separated in this review into four main classes: solution NMR, solid-state NMR, EPR and other methods. Solution NMR methods include the Nuclear Overhauser Effect (NOE) between peptide and membrane signals, residual dipolar couplings and the use of paramagnetic probes, either within the membrane-mimetic or in the solvent. The vast array of solid state NMR methods to study membrane-bound peptide orientation and localization includes the anisotropic chemical shift, PISA wheels, dipolar waves, the GALA, MAOS and REDOR methods and again the use of paramagnetic additives on relaxation rates. Paramagnetic additives, with their effect on spectral linewidths, have also been used in EPR spectroscopy. Additionally, the orientation of a peptide within a membrane can be obtained by the anisotropic hyperfine tensor of a rigidly attached nitroxide label. Besides these magnetic resonance techniques a series of other methods to probe the orientation of peptides in membranes has been developed, consisting of fluorescence-, infrared- and oriented circular dichroism spectroscopy, colorimetry, interface-sensitive X-ray and neutron scattering and Quartz crystal microbalance.

## INTRODUCTION

Membrane-bound proteins and peptides constitute a major class of all expressed proteins and peptides of a genome and are involved in crucial biological processes [1]. Statistical analysis of several genomes of different organisms indicated that 30% of the encoded proteins and peptides are membrane-bound [1-3]. Many naturally occurring peptides bind to biological membranes, examples include peptide hormones, bacterial toxins and antimicrobial peptides [4-6] with the latter being probably the largest and best characterized group of them. A variety of either linear or cyclic antimicrobial peptides have been described to date and they often provide a first unspecific defense mechanism against microbial invasion [4, 7]. Many of these peptides are believed to target microbial cytosolic cell membranes, disrupting them upon interaction. Besides knowing the structure, deciphering the mode of interaction with membranes or membrane-mimetics is necessary to understand their function. While the target of many antimicrobial peptides is the microbial lipid bilayer itself, membrane-binding peptide hormones and bacterial toxins likely act on proteins located in the membrane. Besides membrane-bound peptides, which can be structurally characterized easily by solution NMR spectroscopy, there are an enormous number of membrane-binding proteins. Although the 3D structure determination of soluble proteins by X-ray crystallography and NMR spectroscopy is well-established, far fewer structures of membrane-bound proteins have been solved to date. Structural studies of membrane proteins are considerably complicated by their amphipathic or hydrophobic nature and resulting solubility problems in aqueous buffers. Frequently, their limited stability in the absence of lipids constitutes an additional problem. Less than 2% of all structures in the Protein Data Bank (PDB) are membrane-bound proteins although they account for about 30% of all expressed proteins [3]. Noteworthy, about 45% of all pharmaceuticals target G-protein coupled receptors, which are also a group of membrane-bound proteins [8]. The limited structural information available for these proteins stems either from difficulties in crystallization or their large size, resulting in broad signals and limited spectral dispersion in NMR spectra. While most membrane-bound proteins could not be crystallized or are beyond the current size limit for structural studies by NMR spectroscopy, information about their topology can be obtained by studying the orientation and location of derived shorter peptide sequences in membrane-mimetics. About 80% of residues in membrane-bound proteins are found in α-helices and several membrane associated proteins contain one or more transmembrane α-helices [9]. Knowing the mode of interaction and orientation of these helices with the membrane already goes a long way towards identifying the topology of the whole protein in the lipid bilayer. Membrane-bound peptides are comparatively far easier to handle and can sometimes replace the larger proteins in basic models for folding, interactions and behavior [10]. The localization and orientation of a membrane-bound α-helix is described by three parameters Fig. (**1**) the tilt angle τ, which is defined between the helix axis and the membrane normal (or sometimes the membrane surface), the azimuth (or rotation) angle ρ and the immersion depth d, which can be given for the middle of the helix or a certain residue (e.g. the N-terminal C(α). A number of techniques used for studying the orientation and location of membrane-bound peptides work exclusively on α-helical peptides while others are generally applicable. These methods are mainly based on magnetic resonance spectroscopy (solid state and liquid state NMR and EPR) but other spectroscopic (fluorescence, infrared and oriented circular dichroism) and alternative approaches (colorimetry, Quartz crystal microbalance and interface-sensitive X-ray and neutron scattering) are also frequently used. While all these methods are reviewed here independently, it should be pointed out that they are frequently combined in biophysical membrane-bound peptide studies since they often provide complementary information or can be used on different kinds of membrane-mimetics. It should also be noted that all the methods described in this review work solely on membrane mimetics *in vitro* and to the best of our knowledge there is currently no report of a peptide orientation and/or localization on a biological membrane *in vivo*.

## SOLUTION NMR

Although a number of methodological innovations in the last decades have expanded the size limit of biological macromolecules by solution NMR spectroscopy, the number of structures of proteins or molecular assemblies beyond 35 kDa is still rather small. Therefore, the investigation of membrane-bound peptides has to be restricted to small membrane-mimetic systems. Membrane-mimetic environments typically used for solution NMR studies are micelles, bicelles or small unilamellar vesicles (SUVs) [11-15]. Most commonly SDS (sodium dodecyl-sulfate), DPC (dodecyl-phosphocholine) or DHPC (dihexanoylphosphatidylcholine) have been used as membrane-mimetic due to their commercial availability in deuterated form. SDS is a negatively charged detergent which has been used mainly in studies of antimicrobial peptides as a model for bacterial membranes [16-19]. However, it was shown that it has the potential to influence the secondary structure, especially when studying larger peptides or proteins and that it can induce secondary structures which were not found in other membrane mimetics [20, 21]. On the other hand the zwitterionic DPC or DHPC structurally resemble the components of major eukaryotic biological membranes. They can properly preserve the 3D structure of bound peptides and proteins as well as the catalytic activity of membrane-bound enzymes [22-24]. Even small micelles have radii around 20 Å [*11*] and thus correlation times similar to ~20 kDa proteins. Unlabelled peptides can be NMR spectroscopically assigned up to ~30-35 residues, however, isotopic labeling with ^13^C and ^15^N is necessary for spectral assignment and structure determination of larger peptides or proteins. It should be noted that micelles or SUVs, which are used for solution NMR studies because of their small sizes, possess a large surface curvature, which might in some cases lead to structures or membrane topologies different from the ones found in more relaxed larger vesicles or actual membranes.

Structure determination of small membrane-bound peptides is carried out routinely by high-resolution NMR spectroscopy, since they cannot be studied by X-ray crystallography due to their high flexibility. The structure determination of the peptides bound to micelles can be done on a routine basis using standard homo- or heteronuclear liquid state NMR spectra, but these experiments *per se* reveal nothing about the localization and orientation in the membrane-mimetic. However, a number of techniques have been invented to obtain additional information about the positioning in micelles, bicelles or SUVs.

### Nuclear Overhauser Effect

One of the first solution NMR approaches to obtain information about the localization of peptides and proteins bound to micelles used Nuclear Overhauser Effect (NOE) measurements. NOEs between a micelle-bound protein and the micelle-forming detergent were first described for the integral membrane protein OmpX bound to dihexanoylphosphatidylcholine (DHPC) micelles [25, 26]. NOEs were also used to confirm the topology of the antimicrobial hexapeptide Cyclo(RRWWRF) bound to SDS and DPC micelles [27]. This peptide is bound on the surface of the membrane-mimetics with the aromatic sidechains being oriented towards the micelle center as evidenced by NOEs between aromatic signals and the alkyl chain protons of the detergents. An orientation parallel to the surface was observed for the amphipathic α-helical model class A (apolipoprotein) peptide Ac-18A-NH_2_ bound to DMPC particles [28]. The localization was derived through a series of NOEs, which were found between aromatic peptide protons and alkyl protons of the lipid.

Negative NOEs were found in all these systems, indicating a lifetime of detergent molecules on the peptide surface of more than ~ 0.3 ns. On the other hand the detergent shows only one set of NMR signals implying a fast exchange (on the NMR time scale; i.e. lifetime <~1ms) between protein bound and free detergent molecules. It should also be noted that in order to observe NOEs between the micelle-forming detergents and the bound peptide it is of course necessary to use non-deuterated detergents.

### Residual Dipolar Couplings

Another solution NMR based approach for obtaining information about the tilt and azimuth angle within membrane-mimetics uses residual dipolar couplings (RDCs) [29]. While the direct dipolar coupling of nuclear magnetic moments in highly aligned samples yields coupling constants on the order of several kHz, weak alignment using e.g. bicelles or bacterial phages leads to residual dipolar couplings of a few Hz. The RDC size depends on the nature, distance and angle of an internuclear vector relative to a molecular reference frame. Typically, ^1^H-^15^N bond vectors are used to measure RDCs to obtain long range information on the orientation of these bonds. Regular secondary structural motives lead to periodic variations of RDCs. Plotting RDCs of α-helices versus residue number yields “dipolar waves” which can be fitted to a sinusoid corresponding to the helix periodicity of 3.6 residues per turn [9, 30-32]. Thus, the relative orientations of α-helices in the membrane can be determined, but the immersion depth cannot be obtained. Dipolar waves are very sensitive indicators of any deviation of helices from their ideal geometry, like bends or kinks. Besides yielding the topology of membrane-bound α-helices, dipolar waves can also be used as input for the structure calculation of micelle-bound proteins as shown for the helical membrane protein phospholamban [33]. Inherent flexibility of smaller peptides in a membrane-mimetic leads to reduced RDCs as was found for the pentapeptide Leu-enkephalin bound to the surface of DMPC bilayers [34]. Polyacrylamide gels are typical alignment media which can be used for peptides bound to membrane-mimetics, while filamentous phages cannot be employed due to their incompatibility with detergents [35]. It should be noted that the need for isotopic labeling might prohibit the use of RDCs for some applications.

### Paramagnetic Relaxation Enhancements

One of the most frequently used solution NMR methods for investigating the orientation and localization of peptides bound to membrane-mimetics uses the effect of paramagnetic probes on the peptide itself or tags on the peptide. This approach was pioneered in the group of Wüthrich 30 years ago on the 29-residue peptide glucagon [36]. Through the introduction of 5-, 12- or 16-doxylstearate into dodecylphosphocholine micelles the interior of the micelles is made paramagnetic. When a peptide binds to the micelle its relaxation rates are enhanced by 1/r^6^ with r being the distance between the paramagnetic center and the observed nucleus [37]. The paramagnetic center is furthest inside the micelle when using 16-doxylstearate. It was shown by EPR and NMR spectroscopy that on average one molecule containing the doxyl probe was bound to a micelle of ~50-60 detergent molecules [36]. The paramagnetic effect on glucagon was evaluated qualitatively by determining the relative line broadening for the individual doxyl probes. Using these rough estimates α-helical glucagon was found to be oriented parallel to the surface with the N- and C-terminal residues sticking out of the micelle. Doxyl derivatives are still frequently used for paramagnetic labeling of the interior of micelles. However, it should be kept in mind that micelle forming detergents or lipids are quite flexible in the membrane-mimetic environment and thus the paramagnetic center position is not very well-defined. Therefore, this kind of relaxation enhancements only provides a qualitative picture about the peptide orientation. A more quantitative approach to obtain the orientation and location inside a micelle is the use of the depth dependent partitioning of oxygen towards hydrophobic environments [38-40]. Oxygen is applied at a partial pressure of 20 to 100 atm and produces, due to vastly different oxygen solubility across the micelle, a pronounced paramagnetic gradient inside the micelle. This gradient leads to increased relaxation enhancements in the interior of the micelle and chemical shift perturbations as high as 5 ppm for ^19^F and 0.1 ppm for ^13^C. This approach was applied to a peptide corresponding to the first transmembrane segment of diacylglycerol kinase (DAGK) [38]. To monitor the effects of paramagnetic dioxygen 14 cysteine single point mutants were prepared and chemically conjugated with 3-bromo-1,1,1-trifluoropropanone to introduce ^19^F labels. Differences in chemical shifts observed after applying oxygen at a partial pressure of 100 atm indicated proximity to the hydrophobic micelle interior. Plotting chemical shift changes versus the residue number yielded a wave like pattern with a periodicity of 3.6 residues per turn as expected for an α-helix bound parallel to the surface [38]. However, the introduction of large reporter groups into peptides might in some cases affect their structural und functional properties. Problems could also arise from free cysteine residues present in the peptide or if the membrane contains oxidation-sensitive lipids.

Instead of introducing a paramagnetic probe into the micelle it is also possible to add a paramagnetic compound to the solution surrounding the micelle. In this case the relaxation enhancement affects spins close to the surface of the micelle. Polar residues experience strong relaxation enhancement whereas for hydrophobic residues only weak background enhancement can be detected. Chelate complexes of gadolinium are of common use as water soluble paramagnetic species [26, 27, 41], but also NO-radical bearing compounds like TEMPO [42, 43] or salts of Mn^2+^ or Ni^2+^. A limitation of this approach is that all of these compounds show interactions with some amino acids or even the micelles [44, 45]. Especially the positively charged manganese ions possess high binding affinity to negatively charged areas on the membrane or protein. These interactions and the typically only rough estimates of relaxation enhancements through peak intensity changes allows for distinguishing between surface bound and transmembrane orientations of a peptide only. In contrast gadolinium-diethylenetriamine pentaacetic acid-bismethylamide Gd(DTPA-BMA) is very inert in aqueous solutions. NMR and EPR spectroscopy showed no specific binding of Gd(DTPA-BMA) to micelles or peptides [46, 47]. In addition the stronger paramagnetism of gadolinium versus nitroxide allows the use of lower concentrations to achieve the same relaxation enhancements. If the paramagnetic compound does not penetrate into the micelle, one can describe this situation as rendering the solvent around the micelle paramagnetic. The resulting PRE is the 1/r^6^ integrated volume containing the paramagnetic agent [48]. For a flat surface and to a good approximation for large spherical systems this integrated PRE depends on 1/d^3^ with d being the closest distance to the surface or immersion depth [48]. Adding Gd(DTPA-BMA) to a solution of a peptide bound to a micelle consequently leads to PREs which depend only on the distance to the surface of the micelle. For an α-helical peptide bound parallel to the surface of the micelle this results in a wave with a 3.6 residue periodicity, which we named “paramagnetic relaxation wave” Fig. (**2**) [48]. The tilt- and azimuth angles of the peptide are obtained by least square fitting of experimental PREs to this wave-like function. Using this approach we demonstrated the determination of the orientation of the fifteen residue peptide CM15 in dodecyl-phosphocholine (DPC) micelles [*48*]. If the atomic resolution three-dimensional structure of the peptide is known and a reasonable number of experimental PREs are available it is possible to use this information to obtain both the complete orientation and immersion depth of the peptide in the micelle [47, 49]. Thereby, the PREs, which are proportional to the third power of the immersion depth can be least square fitted to obtain the proportionality constant and the location and orientation within the micelle, in other words the position of the micelle center relative to the peptide. In cases where the number of PREs does not enable a stable least-square fitting the proportionality constant obtained on a larger system can be used. This method was so far used to obtain the complete positioning of the antimicrobial peptides CM15 and maximin H6 in DPC and SDS micelles [18, 47] as well as the transmembrane helix 7 of yeast V-ATPase [47]. The use of Gd(DTPA-BMA) to make the environment paramagnetic has the considerable advantage that it does not require isotopic labeling or any chemical modification of the system under study. However, Gd(DTPA-BMA) is not commercially available in purified form but has to be purified from the MRI contrast agent Omniscan^©^ [48].

## SOLID STATE NMR

In solution NMR spectroscopy, anisotropic interactions of nuclei with the magnetic field are averaged to their isotropic values by rapid molecular reorientation. For the dipole-dipole coupling, chemical shift anisotropy and quadrupolar coupling interactions, the isotropic values are zero, and consequently these interactions are not observed in solution NMR spectroscopy. In solid-state NMR spectroscopy, the anisotropic interactions result in severely broadened lines, thus impeding the resolution of signals from different sites due to signal overlap in spectra of polypeptides [50]. Two different approaches for overcoming this problem have been established: in MAS (Magic Angle Spinning), the anisotropic interactions are averaged by fast spinning of the sample around the ‘magic angle’. This leads to spectra very similar to those obtained by solution NMR spectroscopy. Information on molecule orientation relative to the external magnetic field is lost in this process, but can be regained either by analysis of the spinning sidebands, or by recoupling of weak dipolar couplings using rotor-synchronized pulses. The second approach relies on the use of bilayers, which are uniaxially oriented with respect to the magnetic field. This approach results in a single resonance line from each isotopically labeled site in the polypeptide, while still retaining the orientational information contained in the anisotropic interactions. Generally, the bilayers are oriented with the bilayer normal parallel to the magnetic field direction, so that rotational diffusion around the bilayer normal does not alter the polypeptide’s alignment with respect to the external magnetic field. By far the majority of membrane-bound peptides studied by solid-state NMR are α-helical. The orientation of the helix in the membrane is defined as shown in Fig. (**1**). The azimuth angle (i.e. the rotation angle around the long helix axis) defines the polarity and the tilt angle is the angle between the helix axis and the bilayer normal of the peptide.

### 1D Solid State NMR Spectroscopy

For α-helical peptides the anisotropic chemical shift depends on both the tilt angle and the rotational pitch angle of the polypeptide. The exact values of both are hard to determine by one-dimensional (1D) NMR spectroscopy, as one given shift can correspond to various combinations of values. Still, the anisotropic chemical shift from oriented samples can give hints about the orientation of a polypeptide relative to the membrane: ^15^N chemical shifts of > 180 ppm usually correspond to tilt angles ≤ 20°, while chemical shifts < 100 ppm, are observed for helices that are aligned approximately parallel to the membrane surface [51]. Chemical shifts from 1D NMR spectroscopy together with molecular dynamics simulations have been used to characterize the domain orientations as well as the tilt angle of monomeric phospholamban, which is the main regulator of Ca-ATPase in cardiac muscle [52]. The membrane-disrupting mechanism of two antimicrobial peptides derived from magainin 2 and melittin was investigated by Ramamoorthy *et al.* [53] using ^15^N NMR spectroscopy on aligned bilayers, revealing an orientation approximately perpendicular to the bilayer normal and thereby refuting the barrel-stave mechanism of membrane disruption for these two peptides [53].

### Heteronuclear Dipolar Solid State NMR Spectra

The PISEMA (Polarization Inversion with Spin Exchange at the Magic Angle) experiment correlates ^1^H-^15^N dipolar coupling with ^15^N chemical shifts of membrane peptides and proteins at high resolution [54]. The experiment is based on a flip-flop Lee-Goldburg sequence, which is used to spin-lock the ^1^H spins along the magic angle to suppress the homonuclear (^1^H – ^1^H) dipolar coupling. In PISEMA spectra of oriented and uniformly ^15^N-labeled α-helical membrane peptides, distinct wheel-like patterns are often observed, termed PISA (Polarity Index Slant Angle) wheels Fig. (**3**) [55, 56]. These patterns reveal information on both the rotation and the tilt angle of the peptide. The PISA wheel can be thought of as a projection of the peptide’s helical wheel onto the spectrum plane. The 3.6 residue periodicity of the helical wheel results in a 100° separation of adjacent residues both in the helical wheel as well as in the PISA wheel. It is possible to determine the tilt angle without prior resonance assignments by analyzing the center position, as well as the shape of the PISA wheel. The rotation of the helix about its axis is defined by the distribution of resonances around the PISA wheel, and can be determined with the aid of a single resonance assignment.

When the helix axis is oriented parallel to the bilayer normal (and therefore parallel to the external magnetic field), all amides have an identical orientation relative to the external magnetic field, and therefore all resonances in the PISEMA spectrum overlap. Tilting the helix away from the bilayer normal breaks the symmetry, and leads to dispersion of resonances both in the chemical shift as well as the dipolar coupling domain. The reason for this resolution of resonances lies in the non-colinearity of the ^15^N chemical shift tensor and the ^1^H-^15^N dipolar coupling tensor: the σ33 principal element of the ^15^N chemical shift tensor is rotated by about 17° from the direction of the ^1^H-^15^N bond vector. If this were not the case, the resonances would simply form a straight line instead of the PISA wheel pattern in the spectrum. When the peptide is tilted further away from the membrane normal, some amide NH-bonds are oriented at the magic angle to the external magnetic field, and therefore show dipolar splittings of 0 kHz. For peptides that are tilted by more than about 45°, the amide NH-bonds surpass the magic angle and the sign of the dipolar coupling is inverted. The sign of the dipolar coupling interaction cannot be determined in PISEMA experiments. This results in spectra that seem to be reflected through the 0 kHz axis. Helices that are oriented parallel to the membrane surface show highly overlapped spectra, as all amide NH bond vectors and σ33 components of the chemical shift tensors are nearly orthogonal to the direction of the external magnetic field. Most transmembrane helices studied so far are tilted and therefore show high dispersion of resonances in PISEMA spectra. The study of in-plane helices, on the other hand, often requires the use of 3D correlation spectroscopy.

The PISA wheel approach has been extended to the analysis of the orientation of membrane-associated β-strands [57]. In PISEMA spectra of β-strands oriented parallel to the membrane normal, all resonances overlap. When the peptide is tilted away from the membrane normal, twisted wheel-like resonance patterns, or twisted PISA wheels, appear in the PISEMA spectrum. Contrary to α-helices, PISEMA spectra of β-strands oriented parallel to the membrane surface exhibit good resonance dispersion, owing to the different orientation of ^1^H-^15^N dipolar coupling and ^15^N chemical shift tensors from α-helices. The distribution of resonances in the twisted PISA wheels yields information on both the rotation of β-strand twist as well as on the rotation angle about the β-strand’s own axis.

A PISEMA spectrum was calculated for a β-barrel protein, the outer membrane porin from *Rhodobacter capsulatus*, showing only moderate resolution amongst its resonances [57]. Still, a twisted PISA wheel pattern could be observed, indicating strand tilts of 30° - 60°. This shows that PISEMA spectra of relatively large proteins could give clues about their orientation and topology within the membrane.

In the same study, the PISA wheel approach was also extended to include the use of ^1^H/^15^N heteronuclear correlation (HETCOR) spectra, where wheel-like resonance patterns were observed in simulated HETCOR spectra.

Orientational restraints derived from PISEMA spectra were used in the structure determination of the transmembrane region of the M2 protein H^+^ channel, an α-helical 25 residue peptide from influenza A virus, revealing a 38° tilt relative to the membrane surface [58]. PISA wheels from completely aligned bilayer samples, together with dipolar waves from weakly aligned micelle samples aided in the structure determination of the channel-forming transmembrane domain of virus protein “u” from HIV-1 [59]. The peptide adopts an α-helical conformation with an average tilt of 13°, with a distinctive kink in the center part of the helix. Park and Opella analyzed peptide tilt and rotation angles in aligned samples of bilayers of varying thickness [60]. They demonstrated that the tilt angle, but not the rotation angle of a transmembrane peptide was directly determined by hydrophobic mismatch. Surprisingly, they found that helix kinks tended to disappear in thinner bilayers. Li *et al.* were able to obtain PISEMA spectra of uniformly aligned full-length membrane proteins, with two of the three investigated proteins displaying clear PISA wheel patterns, indicating the arrangement of helices within the proteins [61]. The structure of the membrane protein MerFt, comprising of two transmembrane helices, was solved in magnetically aligned bicelles using PISEMA and SAMMY spectra, complemented by dipolar waves (see below) [62]. The SAMMY pulse sequence, developed by Nezorov and Opella, has the advantage of a reduced frequency offset dependence compared to the PISEMA sequence [63]. The two helices lie nearly parallel to each other and are slightly tilted, with the second, longer helix extending beyond the surface of the bicelle. An interesting study was recently carried out on phospholamban, a regulator of cardiac muscle contractility, using a hybrid solution and solid-state NMR approach [64]. The tilt angle of the transmembrane domain II could be determined to be ≈24° by employing PISEMA spectra to gather orientational restraints for structure determination.

### Dipolar Waves

Plotting the ^1^H-^15^N dipolar coupling versus residue number yields a one-dimensional dipolar wave as an extension of the two-dimensional PISA wheels [9, 32]. The dipolar couplings can either stem from solid-state NMR spectroscopy of highly aligned samples, or from solution NMR spectroscopy of weakly aligned samples. The dipolar couplings show a periodic wavelike variation with the sequential residue number which reflects the 3.6 residue periodicity of the α-helix. Similarly to PISA wheels, the dipolar wave pattern is a result of the non-colinearity of the long helix axis and the v║ component of the ^1^H-^15^N dipolar coupling tensor, which coincides with the NH bond vector. The NH bond vector in an ideal α-helix is tilted away from the helix long axis at an angle of 15.8°. Fitting of the experimentally determined dipolar couplings as a function of residue number to sinusoids of periodicity 3.6 yields both the tilt angle and the polarity (rotation) of the helix. For completely aligned bilayer samples, the results are unambiguous, while data from weakly aligned bilayer samples result in 4^N-1^ possible orientations for N helices. However, these ambiguities can be resolved by comparison to a differently aligned sample [65]. In magnetically aligned fd filamentous bacteriophages particles the structure and orientation of the coat protein were determined using both PISA wheels and dipolar waves [66]. The data revealed a distinct kink in the helix, which was in contrast to a prior fiber diffraction study which had suggested a gently curved α-helix. It is a remarkable feature of the dipolar wave approach that deviations from the ideal conformation along the helix become apparent from the fit of the experimental data [31]. Dipolar waves and PISA wheels were also used in the structure determination and localization of the coat protein in Pf1 bacteriophage [67].

### The Geometric Analysis of Labeled Alanines

The GALA (Geometric Analysis of Labeled Alanines) method [68, 69] relies on orientation information from quadrupolar splittings of deuterium atoms in CD_3_-labelled alanines in α-helices. Like the dipolar coupling, the quadrupolar coupling has a 3cos^2^(θ-1) dependence, where θ is the angle between the external magnetic field and the carbon-deuterium bond. The magnitude, but not the sign of the quadrupolar splitting can be determined, and typical values lie around 150 – 170 kHz. The quadrupolar splitting depends on the position of the alanine sidechain relative to the helix, as well as the orientation of the helix within the membrane. The first is defined by the angle ε║ of the C_α_-C_β_ bond with the helix direction, and the angle ε┴ with the plane perpendicular to the helix direction. The helix orientation is defined by the rotation angle ρ, which describes the rotation about the helical axis, and the tilt angle τ between the helix axis and the membrane normal. The orientation of the membrane-spanning peptide WALP19 was determined using this approach, yielding a small tilt angle of 4° relative to the membrane normal [69]. The tilt angle was found to be rather insensitive to bilayer thickness or hydrophobic mismatch, indicating that the tilt angle is determined by intrinsic characteristics of the peptide. Similar results were found for the longer WALP23 peptide and its analogues [70, 71]. Employing the GALA method, the formation of an antimicrobial heterodimer between the peptides PGLa and magainin was observed by monitoring the change in tilt angle of PGLa upon addition of magainin [72]. The GALA and PISA wheel methods have been compared to each other by analyzing the transmembrane peptide GWALP23, resulting in close agreement between results of both methods [73].

### Magic Angle Oriented Sample Spinning

Magic Angle Oriented Sample Spinning (MAOSS), introduced in 1998, combines benefits of static oriented solid-state NMR spectroscopy and magic angle spinning NMR spectroscopy [74]. While often employed to obtain very high resolution spectra, or for investigation of membrane alignment, MAOSS can also provide information about the distribution of orientations of polypeptides within a sample. When a sample is rotated at the magic angle, the anisotropic chemical shift interaction is averaged, and therefore solution-like spectra with single peaks at the isotropic chemical shift frequency are obtained for every amide site. The orientational information in the anisotropic chemical shift interaction is lost in the process. However, when spinning the sample at speeds lower than the size of the chemical shift anisotropy, spinning sidebands occur. If all orientations in the sample are equally distributed, the sidebands have an envelope that corresponds to the shape of the powder spectrum. In partially ordered samples, the spinning sidebands contain information about the degree of alignment within the sample, as well as the tilt angle between the polypeptide and the membrane normal. Most of the MAOSS studies reported in the literature have been carried out on larger membrane proteins [75-77], but the technique is of course equally well suited for smaller polypeptides.

### Other Solid State NMR Techniques

An experimental scheme has been proposed which allows determination of polypeptide orientation for unoriented samples under fast spinning at the magic angle (i.e., not relying on the analysis of spinning sidebands) using recoupling sequences [78]. The orientation of the antimicrobial peptide tachyplesin I was determined by employing a REDOR pulse sequence [79] for recoupling of dipolar coupling between ^13^C nuclei in the peptide and ^31^P nuclei in the bilayer, allowing for determination of ^13^C-^31^P distances [80]. Additionally, the depth of insertion of the peptide was inferred from two-dimensional (2D) ^1^H spin diffusion experiments, where spin diffusion from lipid and water protons was detected through the peptide ^13^C signals. A similar approach was employed by Tang *et al.* in their study of the cationic antimicrobial peptide PG-1 [81]. An interesting technique, which allows the determination of membrane alignment of helical peptides in non-oriented samples, was described [82]. The technique employs rotational diffusion of membrane-associated peptides around the membrane normal, which is sufficiently fast to cause motional averaging of the chemical shift interaction. The resulting shift is a function of the axis of motional averaging, and can therefore be used to determine the position of the peptide axis relative to the membrane. Paramagnetic Mn^2+^ ions have been used to determine distances semi-quantitatively between ions in solution, and the membrane-bound antimicrobial peptide protregrin-1 via distance-dependent paramagnetic relaxation enhancements (PREs) [83]. The PRE effect on the peptide nuclei is compared to that of the lipids, allowing for an estimate of distance from the membrane surface. The orientation of the cyclic β-sheet peptide gramicidin S containing a ^19^F-labeled amino acid was determined by analyzing the orientation of the ^19^F chemical shift tensor relative to the membrane normal [84].

For all solid-state techniques mentioned so far an immobilized helix has been described. However, in a liquid crystalline membrane, rotational diffusion around the membrane normal as well as fast rotation around the helix axis takes place. These motions result in a scaling of the chemical shift and dipolar anisotropies depending on the orientation of the peptide in the membrane and the membrane normal with respect to the applied magnetic field. For accurate orientational parameters these degrees of freedom have to be considered [51].

## EPR SPECTROSCOPY

Paramagnetic agents have also been used extensively for probing peptide membrane interactions by electron paramagnetic resonance spectroscopy. Similar to the above-mentioned solution NMR technique, EPR has been used to monitor the effect of either micelle bound oxygen or soluble nickel ethylenediaminediacetate on the linewidth or relaxation time of spin-labeled membrane-binding peptides [85]. The spin label was attached by covalent bonding of e.g. methanethiosulfonate nitroxide radicals to cysteines which were introduced into the peptide during solid phase peptide synthesis [86]. To obtain the actual immersion depth from the saturation the α-helical transmembrane peptide WALP was used as a ruler [85]. Each amino acid of this 23-residue peptide was individually replaced by cysteine and subsequently a spin label introduced. Using the known orientation of this peptide in membranes, the immersion depth of each residue could be calculated. This information can then be used to obtain the orientations of other unknown peptides. Individual amino acids need to be replaced by cysteines consecutively to obtain the position of a peptide in the membrane. The measured linewidth reports on the collision rate between the spin-label and surrounding paramagnetic probe. For α-helical peptides the tilt and azimuth angle can be obtained from the resulting wave-like behavior of the saturation parameter of the spin-label as a function of position within the peptide. This approach was applied, for example, to determine the orientation of the designed antimicrobial peptide CM15 in phosphatidylethanolamine and phosphatidylglycerol vesicles [86].

Another elegant EPR approach towards obtaining the topology of peptides in bilayers exploits the anisotropic hyperfine tensor of the nitroxide spin label 2,2,6,6-tetra-methylpiperidine-1-oxyl-4-amino-4-carboxylic acid (TOAC) [87-89]. This agent, which needs to be introduced by chemical synthesis into the studied peptide, is a rigid paramagnetic probe whose hyperfine splitting highly depends on the orientation in the magnetic field Fig. (**4**). The tilt and azimuth angle of α-helical peptides can be derived from a wave like pattern obtained by introducing TOAC probes at different positions along the peptide chain and measuring the hyperfine coupling in aligned bilayers for each sample [90]. Alignment in the magnetic field can be achieved by e.g. paramagnetically doped bicelles or by mechanical orientation.

Compared to the above-mentioned NMR techniques these EPR methods have the considerable advantage of being applicable to much smaller amounts of peptide due to the intrinsically higher sensitivity of EPR. However, the peptides to be studied need to be chemically modified which is not only laborious but might also influence the behavior in a hydrophobic environment.

## OTHER METHODS

Besides these magnetic resonance techniques a few other methods for the determination of the orientation and/or localization of membrane-bound peptides have been reported and will be described briefly in the following. Most of these methods are far more sensitive than the above-mentioned magnetic resonance techniques, in particular NMR, but provide less specific information on the orientation and localization of membrane-bound peptides. All the methods presented below do not suffer from an intrinsic size limit (like e.g. solution NMR) and are therefore typically used on larger membrane-mimetics (e.g. vesicles).

## FLUORESCENCE METHODS

Fluorescence techniques provide a sensitive approach towards determining the approximate position and orientation of lipid associated biomolecules [91]. Labeling of either the peptide of interest or the surrounding lipid with a fluorescent tag is necessary for the application of this method unless the peptide under investigation contains at least one tryptophan. Due to the high sensitivity compared to magnetic resonance techniques, fast kinetics of peptide insertion into the membrane can also be investigated.

### Tryptophan Fluorescence

Membrane-bound peptides often possess intrinsic fluorescence caused by aromatic amino acids, with tryptophan residues giving by far the highest quantum yield [92, 93]. Tryptophan fluorescence is strongly influenced by other amino acids or lipids in the vicinity. Since tryptophan is a rather rare amino acid and many peptides do not contain more than one or two Trp residues, the fluorescence spectra can often be analyzed with residue resolution. Tryptophan fluorescence is especially sensitive to the polarity of the surroundings, and can therefore be used to determine binding affinities of Trp-containing peptides to membrane mimetics. Upon interaction with a hydrophobic environment the tryptophan fluorescence emission is shifted towards shorter wave-lengths (blue-shift) and decreases in intensity [92, 94]. The degree of blue shift can be correlated with the membrane insertion depth. Besides this polarity related changes of tryptophan fluorescence, additional information on the localization of the tryptophan can be obtained by using either aqueous or membrane-embedded quenchers. Typical aqueous quenchers are iodide ions or acrylamide, while e.g. brominated or methyl-coumarin labeled phosphocholines can be used to quench the fluorescence in the hydrophobic environment Fig. (**5**) [95, 96]. Examples for the use of tryptophan fluorescence for peptide-membrane interactions are an early study by de Kroon *et al.* [95] on a series of designed penta-, and hexapeptides on SUVs using a series of aqueous and membrane-embedded quenchers, or a report on penetratin membrane insertion and translocation upon its interaction with negatively charged lipids [94]. The kinetics of membrane-insertion of magainin 2 were monitored by FRET (Förster resonance energy transfer) between tryptophan and p-cyano-L-phenylalanine [97].

### Solvent Relaxation

Solvent relaxation (SR) refers to the transient process of solvent reorganization in response to a sudden change in charge distribution of a fluorescent dye via electronic excitation [98]. Therefore, the frequency maximum of fluorescence emission spectra is recorded time resolved. In phospholipid bilayers this relaxation occurs usually on the ps to ns timescale depending on the lipid composition and the localization of the dye molecule [99]. The fluorescent probe is either inserted into the lipid membrane bilayer or covalently bound to the fatty acid chain or the phospholipid. The SR depends on the viscosity and polarity of the lipids (and the probe itself) at a certain position in the bilayer. The localization of the fluorescent part of the dye in the membrane has to be known to calculate the influence a peptide has on the emission. Depending on where and how deep a peptide inserts into the bilayer the emission frequency maximum difference (the Stokes shift) varies. Solvent relaxation is expected to occur at a slower rate when a peptide is inserted deeper into the bilayer, although this can vary due to changes in the mobility of phospholipid headgroups or location changes of water molecules around the fluorescent dye [99]. Sheynis *et al.* [100] used this technique to corroborate the importance of electrostatic interactions in the binding of antimicrobial peptides to membranes by showing a deeper insertion of cationic peptides into a negatively charged bilayer compared to insertion in membrane-mimetics consisting of zwitterionic lipids.

## INFRARED SPECTROSCOPY

Infrared spectroscopy has been used for quite some time to obtain information on the secondary structure of proteins and peptides mainly through the so-called amide I band (the C=O stretching vibration around 1650 cm^-1^) [101]. The secondary structure of membrane-bound peptides and proteins can by very conveniently analyzed by ATR (attenuated total reflection) IR spectroscopy of thin hydrated films of peptides in membranes [102]. While IR spectra of soluble proteins are dominated by water signals, spectra of membrane proteins can be recorded in hydrated lipid bilayers, where water opacity is much less of a problem [103]. In addition to the structure also the orientation of regular secondary structural elements relative to the lipid membrane plane can be determined by ATR-IR on thin hydrated lipid films [104]. By using infrared light polarized at 0° and 90° relative to the ATR plane, it is possible to obtain information on the orientation of the main transition dipole moment of the peptide amide C=O bonds. In an α-helix these vectors lie almost parallel to the helix axis, while in an antiparallel β-sheet the polarization is perpendicular to the strand axis. The polarization is typically expressed as the absorbance of 90°/absorbance of 0° polarized light which is called the dichroic ratio. From this ratio the mean angle of the C=O bond relative to the ATR surface can be calculated according to Fringeli and Günthard [105]. For the interpretation of these data one has to keep in mind that, if the peptide samples different orientations, the calculated geometry is an average. A more accurate picture of a distribution of orientations of membrane-bound peptides can be obtained by a combination of ATR-IR with sum frequency generation (SFG) vibrational spectroscopy [106]. A SFG spectrum is generated by spatial and temporal overlapping of beams of visible light and tunable IR. The resulting sum frequency beam is detected as a function of the IR frequency. When the IR frequency is in resonance with a molecular vibration, the intensity of the sum frequency peak is enhanced. Combining SFG and ATR-IR results in more measureable parameters and therefore a more accurate distribution of orientations can be obtained. The amide I band which is used for this analysis is rather broad and individual amide bonds are not resolved. This problem can be overcome by employing selective isotope labeling. Isotopically edited IR, where single carboxyl groups are labeled by ^13^C, ^18^O or ^2^H provides site specific C=O orientation information inferred from signals shifted to a less crowded area of the spectrum [102]. The orientation in a membrane has been determined for a series of peptides by Bechinger *et al.* [104]: magainin 2, the designed antimicrobial peptide LAH_4_, the transmembrane hydrophobic regions of glycophorin and the model peptide hΦ20. In this study the orientations within the membranes were correlated with results from solid-state NMR spectroscopy so that model-dependent parameters necessary for the analysis of ATR-IR spectra could be quantified. A twofold distribution of orientations of the antimicrobial peptide melittin in a membrane bilayer was found using a combination of SFG and ATR-IR by Chen *et al.* [106]. In the group of Arkin the residue-specific orientations of peptides were studied by introducing series of individual site-specific ^13^C=^18^O labels along peptides [107]. These data were accurate enough to detect a kink in the transmembrane helix of T-cell receptor CD3-ζ.

## COLORIMETRY

Amphiphilic diacetylene monomers such as pentacosadiynoic acid, which can be polymerized, typically appear blue due to their large conjugated network. A variety of environmental changes (e.g. temperature , pH or surface pressure) are accompanied by a color change, believed to be caused by an irreversible structural transition of the conjugated polymer backbone. Together with phospholipids, mixed phospholipid/PDA vesicles could be produced which act as membrane-mimetics. A supramolecular assembly of polydiacetylene (PDA) with conjugated lipids undergoes a color change from blue to red when binding to membrane-active biomolecules [108, 109]. The color change occurs when the length of the PDA lipid backbone is reduced (conjugated length change) probably due to a loss in planarity, resulting in other wavelengths being absorbed. The method allows for fast and easy screening of peptides interacting with phospholipid/PDA vesicles [109]. Peptides binding differently to membranes induce different degrees of color transitions. The deeper the peptide penetrates this membrane-mimetic the stronger the color change. Sheynis *et al.* [100] employed colorimetry to compare the depth of peptide immersion of melittin, magainin and the synthetic hydrophobic peptide KAL.

## ORIENTED CIRCULAR DICHROISM SPECTROSCOPY 

Molecules containing chiral chromophores absorb right- and left-handed circularly polarized light. In circular dichroism spectroscopy the difference between the absorptions of these two electromagnetic waves are monitored as a function of wavelength. CD spectroscopy, more precisely UV-CD, is used to study the secondary structure of proteins or peptides and its changes in different environments, e.g. temperature, pH, ligands, or denaturants [110]. Identification of structural elements and structural changes is possible due to the fact that each secondary structure shows a specific CD signature. The orientation of α-helical peptides in a multilayer lipid membrane can be determined by CD spectroscopy using light transmitted perpendicular and under oblique angles relative to the membrane plane [111, 112]. Spectral artifacts due to dielectric interfaces, birefringence and linear dichroism need to be removed, typically by subtracting a spectrum under the same conditions in the absence of the peptide. This method has for example been used to determine the orientation of the α-helical peptide alamethicin in diphytanoylphosphatidylcholine multilayer [112], to determine the effect of the cationic antimicrobial peptide novicidin on membrane integrity [113] or to study the effect of membrane composition on the orientation of the antimicrobial peptides aurein 2.2 and 2.3 in mixed phosphatidylglycerol/phosphocholine [114] membranes to name only a few.

## INTERFACE-SENSITIVE X-RAY AND NEUTRON SCATTERING

While the three-dimensional structure determination of proteins by X-ray crystallography has been carried out successfully on thousands of proteins and can be considered routine, far less structures of membrane-bound proteins and peptides could be determined. Crystal structures are available for a few membrane-bound peptides, which were crystallized from water or co-crystallized using organic solvents as membrane-mimetics [115, 116]. Peptides bound to actual membrane-bilayers or micelles cannot be studied by X-ray crystallography. However, by in-plane X-ray and especially neutron scattering, structural features in membranes can be detected when the neutron or electron density is different compared to pure lipid bilayers [117]. For example, pores in a membrane can be monitored by incorporating D_2_O into the membrane, resulting in a modified contrast and thus scattering intensity. Information about the peptide structure can be inferred from the low signal of the molecular form factors, if detectable. In-plane ordering is manifested by super-structure peaks in the small-angle region of the scattering curve. A change of the membrane geometry upon peptide insertion contains information about the bound peptide in the bilayer. However, it is not possible to acquire direct information about the peptide immersion by this technique. Aligned multiple lipid layers, being able to amplify the scattering signal, are convenient model systems to study insertion and conformation of membrane-bound peptides [117]. For instance, the peptide/lipid ratio dependent channel formation of the antimicrobial peptide magainin was investigated by neutron scattering in multilayers, identifying the transition of peptide orientation from parallel alignment to a vertical, membrane-spanning orientation to be the critical part in the channel forming process, ultimately leading to cell lysis [118]. Recently, a heavy-atom spin-label allowing the localization of membrane-bound peptides by X-ray scattering has been introduced [119]. The chemically synthesized 5-diiodo-allylglycine can be incorporated into a peptide by Fmoc-based solid-phase peptide synthesis. The position of the amino acid replaced by the heavy-atom label can be pin-pointed in the membrane through its increased electron density and therefore higher contrast for X-ray scattering. Due to the weak scattering properties of biomolecules, peptide scattering curves are usually obtained by using high intensity beams generated by synchrotron radiation sources.

## QUARTZ CRYSTAL MICROBALANCE

A QCM (or QCM-D for Quartz crystal microbalance with dissipation) consists of a piezoelectric quartz crystal functioning as mechanical resonator. Disturbance of the resonance, due to small changes in mass, results in shifts of the recorded resonance frequency. Local mass changes as a function of distance to the sensor surface can be detected by recording overtones of the natural frequency of the quartz sensor chip. Consequently, not only mass, film thickness, and viscosity can be measured, but it is also possible to localize disruptive membrane peptide interactions [120]. If different order overtones are similar (indicating the same mass) and the peptide has the same structural characteristics across the whole membrane, a transmembrane orientation can be inferred. By analyzing overtone effects, three antimicrobial peptides were shown to behave differently upon DMPC membrane interaction: Caerin 1.1 adopted transmembrane orientation, maculatin 1.1 exhibited concentration dependent membrane disruption, while aurein 1.2 aligned itself parallel to the bilayer surface [120]. Recently, the reversibility of membrane-binding of the cationic antimicrobial peptide novicidin [113] was shown with QCM.

## CONCLUSION

To understand the function of membrane-bound peptides, either naturally occurring peptides or fragments of membrane proteins, knowing the location and orientation in the membrane or a membrane-mimetic is often essential. Meanwhile, a large number of methods, which have been reviewed here are available to probe these parameters. The majority uses magnetic resonance (solution and solid-state NMR and EPR) although a wide area of alternative techniques, like CD, fluorescence, colorimetry or QCM is also available. The information obtained by the individual approaches is often complimentary and therefore the application of several methods on a specific question is recommended.

## Figures and Tables

**Fig. (1):**
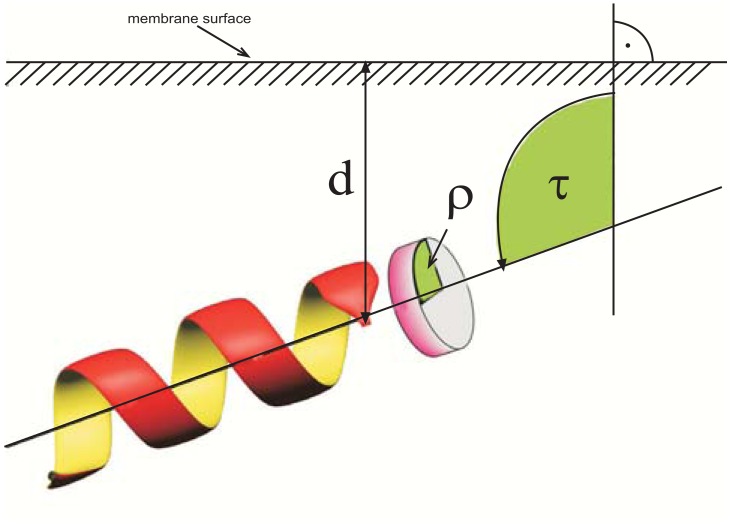
Three parameters are needed for the positioning of a membrane- bound peptide: The tilt angle ι, the azimuth (or rotation) angle ρ and the immersion depth. Please note that sometimes the tilt angle is defined between the helical axis and the membrane surface.

**Fig. (2):**
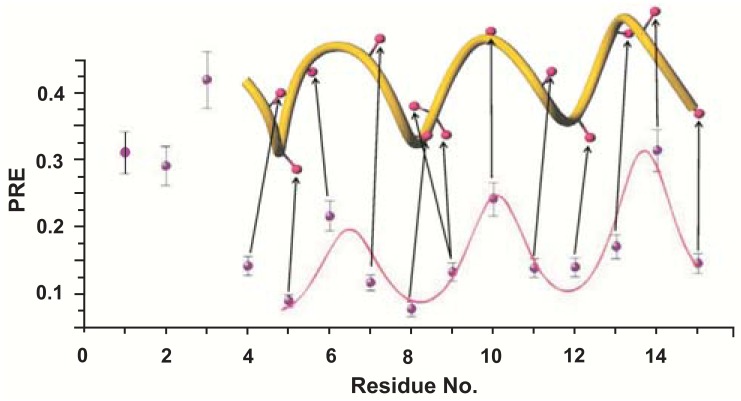
Relaxation enhancements in a paramagnetic environment (PRE) as a function of residue number are shown for the antimicrobial peptide CM15 together with the backbone ribbon diagram of its NMR structure in DPC micelles. The experimental values can be fitted to a “paramagnetic relaxation wave” (pink) to obtain the tilt and azimuth angles. Reproduced with permission from [48].

**Fig. (3):**
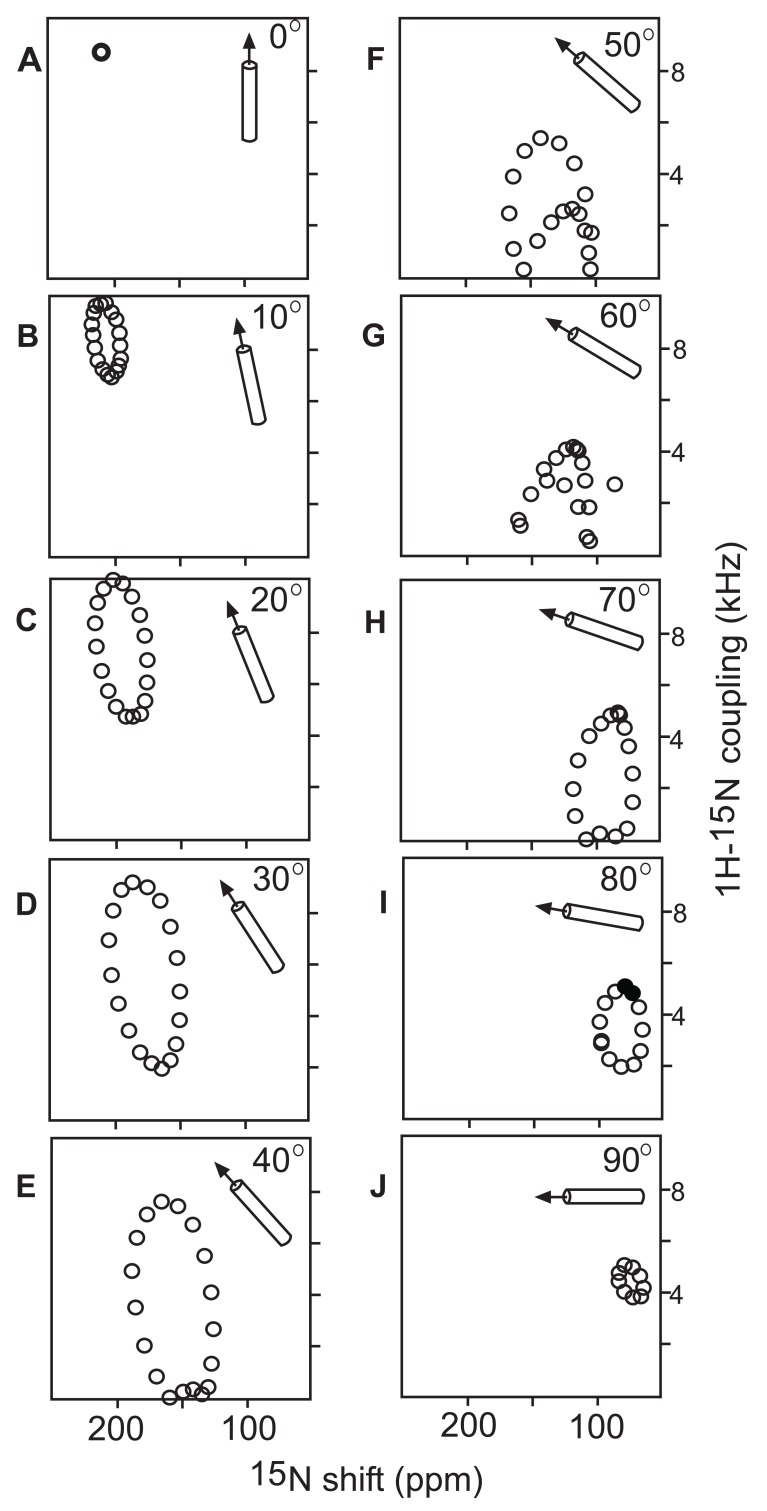
PISA wheels calculated for a 19-residue α-helical peptide for tilt angles ranging from 0 to 90° (relative to the bilayer normal). Reproduced with permission from [55].

**Fig. (4):**
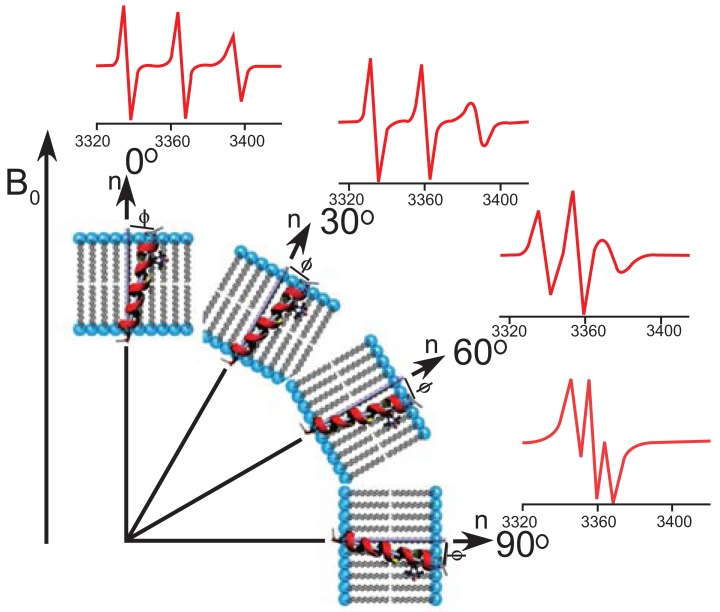
EPR spectra (X-band) of a TOAC spin label attached to the transmembrane helix M2δ of the acetylcholine receptor as a function of the orientation of the bilayer normal relative to the magnetic field. Changes in the hyperfine splitting allow for a direct determination of the tilt angle. Reproduced with permission from [88].

**Fig. (5):**
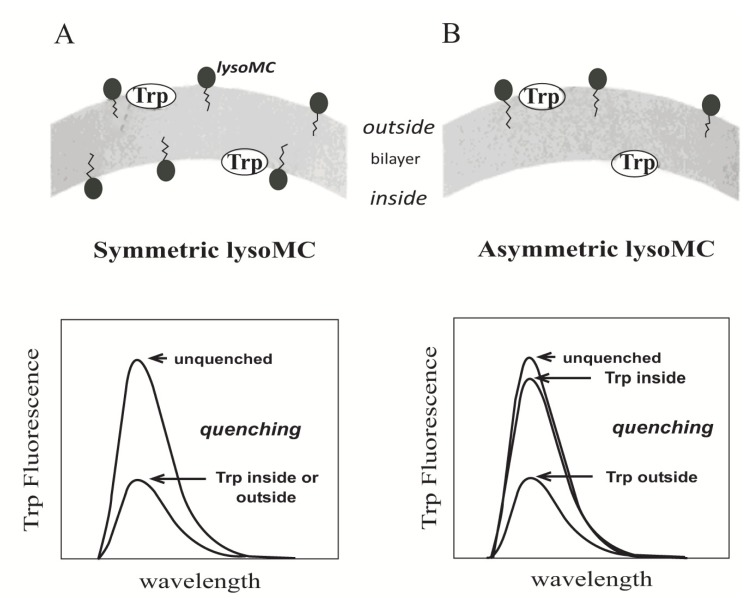
Schematic representation of the effect of the lipid lyso-MC inserted either symmetrically (**A**) or on the outer surface (**B**) of LUVs on the tryptophan fluorescence. Proximity of lysoMC results in fluorescence quenching. Reproduced with permission from [96].
